# Error in Text

**DOI:** 10.1001/jamanetworkopen.2018.3588

**Published:** 2018-09-14

In the Invited Commentary by McCullough and Flowers titled “Identifying and Addressing Disparities in Survival Outcomes for Rural Patients With Cancer,” published August 17, 2018, in *JAMA Network Open,*^1^ there were errors in the fifth paragraph. The paragraph should read as follows: “Finally, the current study was centered on the first 5 years after registration to focus on cancer-related and treatment-related survival. Although the study authors allowed for time variation between 1 and 10 years to account for differential prognostic patterns by cancer site, it remains unclear whether differences in survival between rural and urban patients are observed beyond these terms. Because of improvements in screening and treatment, many cancer survivors are expected to live well beyond 5 years following a cancer diagnosis. Thus, disparities in survival between rural and urban patients farther along in the postdiagnostic period also are of interest for future planning and resource allocation.” This article was corrected.
